# Putting the Biological Species Concept to the Test: Using Mating Networks to Delimit Species

**DOI:** 10.1371/journal.pone.0068267

**Published:** 2013-06-20

**Authors:** Lélia Lagache, Jean-Benoist Leger, Jean-Jacques Daudin, Rémy J. Petit, Corinne Vacher

**Affiliations:** 1 INRA, UMR1202 BIOGECO, Cestas, France; 2 Univ. Bordeaux, BIOGECO, UMR 1202, Talence, France; 3 INRA, UMR 518 MIA, Paris, France; 4 AgroParisTech, UMR 518 MIA, Paris, France; Universita' del Piemonte Orientale, Italy

## Abstract

Although interfertility is the key criterion upon which Mayr’s biological species concept is based, it has never been applied directly to delimit species under natural conditions. Our study fills this gap. We used the interfertility criterion to delimit two closely related oak species in a forest stand by analyzing the network of natural mating events between individuals. The results reveal two groups of interfertile individuals connected by only few mating events. These two groups were largely congruent with those determined using other criteria (morphological similarity, genotypic similarity and individual relatedness). Our study, therefore, shows that the analysis of mating networks is an effective method to delimit species based on the interfertility criterion, provided that adequate network data can be assembled. Our study also shows that although species boundaries are highly congruent across methods of species delimitation, they are not exactly the same. Most of the differences stem from assignment of individuals to an intermediate category. The discrepancies between methods may reflect a biological reality. Indeed, the interfertility criterion is an environment-dependant criterion as species abundances typically affect rates of hybridization under natural conditions. Thus, the methods of species delimitation based on the interfertility criterion are expected to give results slightly different from those based on environment-independent criteria (such as the genotypic similarity criteria). However, whatever the criterion chosen, the challenge we face when delimiting species is to summarize continuous but non-uniform variations in biological diversity. The grade of membership model that we use in this study appears as an appropriate tool.

## Introduction

According to the biological species concept, the ability to interbreed (i.e. interfertility) is a defining property of species [1]. Yet, to our knowledge, the interfertility criterion has never been used to delimit species on the basis of mating events observed under natural conditions. Only artificial crosses have been used for this purpose, including in fungi (e.g. [2]), plants [3], or insects [4]. However, this approach has been criticized (e.g. [5,6]) because artificial crosses bypass some pre-mating barriers to hybridization: mating events observed under artificial conditions might not reflect what would naturally occur. Hence, to date, there is no satisfactory example of the use of the interfertility criterion to delimit species. In fact, the methods used most frequently for species delimitation are not derived from the well-known biological species concept but are derived from other concepts such as the phylogenetic species concept, the genotypic species concept and the morphological species concept. Species definitions according to these concepts and possible associated criteria for species delimitation are listed in Table 1.

**Table 1:** Major species concepts with associated possible criterion for species delimitation.

**Species concept**	**Species definition according to this concept**	**Possible criterion for species delimitation derived from this definition**	**Possible method of species delimitation using this criterion**	**First application of this method at the study site**
Biological species concept	Species are “*groups of actually or potentially interbreeding natural populations, which are reproductively isolated from other such groups*”[1]. According to Hausdorf [17], “natural populations” can be replaced by “individuals” in this statement without change of meaning.	Higher natural interfertility between individuals within than among species	Clustering of the network of natural mating events between individuals using Continuous Stochastic Block Model (C-SBM) [24].	this study
Phylogenetic species concept	A species is “*a diagnosable cluster of individuals within which there is a parental pattern of ancestry and descent, beyond which there is not, and which exhibits a pattern of phylogenetic ancestry and descent among units of like kind*” [29].	Higher genetic relatedness between individuals within than among species	Clustering of the network of relatedness relationships between individuals using C-SBM [24].	this study
Genotypic species concept	A species is a “*genotypic cluster of individuals that can overlap without fusing with its siblings*” [17,52]	Higher genotypic similarity between individuals within a species	Clustering of the individuals based on their multilocus genotype with STRUCTURE [50]	Guichoux *et al.* 2012 [23]
Morphological species concept	Species are “*the smallest detected samples of self-perpetuating organisms that have unique sets of characters*” [53,54].	Higher morphological similarity between individuals within than among species	Clustering of the individuals based on several morphological traits with a factorial discriminant analysis [55].	Bacilieri *et al.* 1996 [26]

**Figure 1:**
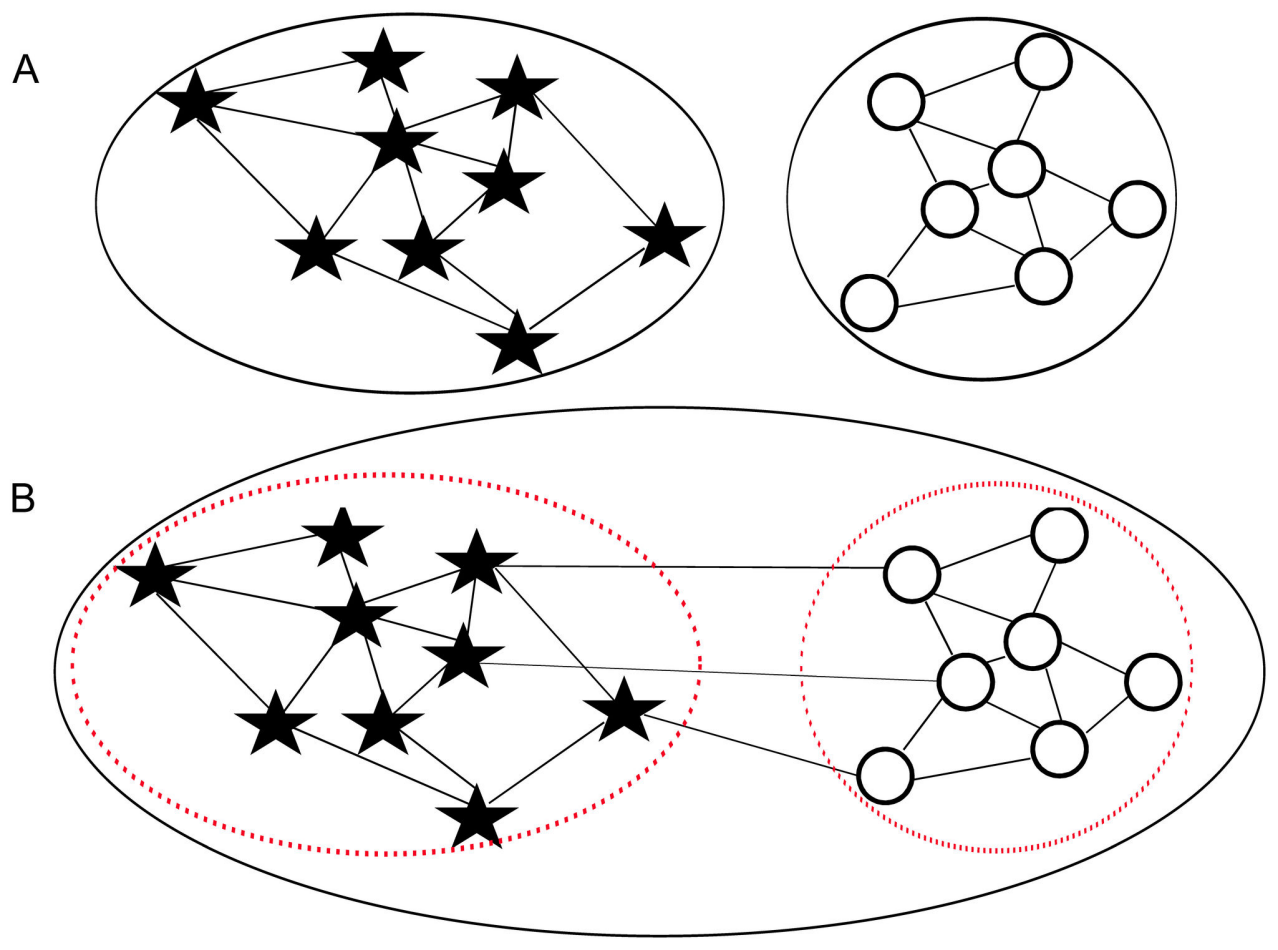
Example of mating networks with species boundaries. Each node of the network, represented by a black star or a white circle, is an individual. Each link of the network, represented by a thin black line, corresponds to a mating event between two individuals. In A, there is no mating event between the two groups of individuals whereas in B, a few mating events occur between groups. Species boundaries according to a strict application of the biological species concept are indicated by a continuous thick black line. Species boundaries according to a relaxed interpretation of the biological species concept, allowing interspecific hybridization, are indicated by a broken red line. In network theory, the continuous black line delimits the connected components of the network whereas the broken red line delimits communities.

One potential method of species delimitation based on the interfertility criterion is the analysis of mating networks. Mating networks represent mating events between individuals [7]. Nodes of the network represent the individuals and links connect the individuals between whom mating events have occurred. Applying methods of network clustering [8–10] to mating networks should allow the identification of subsets of strongly interconnected nodes that correspond to species. If the biological species concept is strictly interpreted, then a species should correspond to a connected component of the mating network (Figure 1A). A connected component is a subset of nodes within the network that are directly or indirectly connected but are not connected to nodes not contained in the subset. According to a relaxed biological species concept, which allows for some level of hybridization between species [11–13], a species should correspond to a community in the mating network (Figure 1B). Communities are subsets of nodes with a high density of links within the group and a lower density of links between different groups [8]. It is in this latter case, when species hybridize, that species delimitation based on the interfertility criterion is particularly challenging and network analysis may be particularly useful.

The idea of analyzing mating networks to delimit species according to the biological species concept was proposed more than 40 years ago by Sokal and Crovello [14] but it does not appear to have been put into practice. Building a mating network is indeed a difficult task as it requires a very large data set of mating events collected under natural conditions. The species should be sympatric and have semi-permeable reproductive barriers so that the issue of species delimitation is relevant. Furthermore, the species should be not only outcrossing (with a low selfing rate) but also highly polygamous and have multiple offspring per generation so that actual mating events are representative of potential mating events between individuals at a given time [15–17]. If such data were available, would the analysis of mating networks be an effective method to delimit species based on the interfertility criterion? Would the boundaries between species be the same as those obtained using other species delimitation criteria?

To answer these questions, we investigate the congruence between four methods of species delimitation, derived from the biological, morphological, genotypic and phylogenetic species concepts (Table 1), by applying them to two hybridizing tree species living in sympatry. The study site is a 5 ha mixed stand of *Quercus* *robur* and *Q* *. petraea* comprising 298 adult trees originating from natural regeneration [18]. As many other closely related plant species [19], these two oak species hybridize under natural conditions [20], including in the studied stand [21–23]. To delimit species according to the interfertility criterion, we analyze the network of observed natural mating events between pairs of adult trees by using a method of network clustering. Each node of the mating network corresponds to an adult tree and each link corresponds to at least one mating event between two trees. To cluster individuals, we selected among available methods of network clustering [8–10] the Continuous Stochastic Block Model (C-SBM) recently introduced by Daudin *et al*. [24]. C-SBM synthesizes the heterogeneity of a real network by producing a simplified version of the network composed of a few virtual nodes, called extremal hypothetical nodes (EHNs). Unlike many methods of network clustering, which assume that each node belongs to only one group, C-SBM allows nodes to exhibit mixed connectivity behavior by assuming that each node of the real network is a mixture of the EHNs. This method is thus particularly suited to our study. Indeed, because the two previously identified oak species [23,25] are known to hybridize [22], we expected to find some individuals with a mixed reproductive behavior, i.e. breeding with both species. The same method was used to delimit species based on genetic relatedness between individuals. In that case, each node of the network corresponds to an adult tree and links connect the individuals that are considered to be related based on their genotype. Finally, we compare individual assignments obtained by analyzing the mating network and the relatedness network with those previously obtained in the same study site using criteria of morphological and genotypic similarities [23,25]. We then discuss how to summarize continuous but non-uniform variations in biological diversity.

## Results

### Species Delimitation based on Interfertility

**Figure 2:**
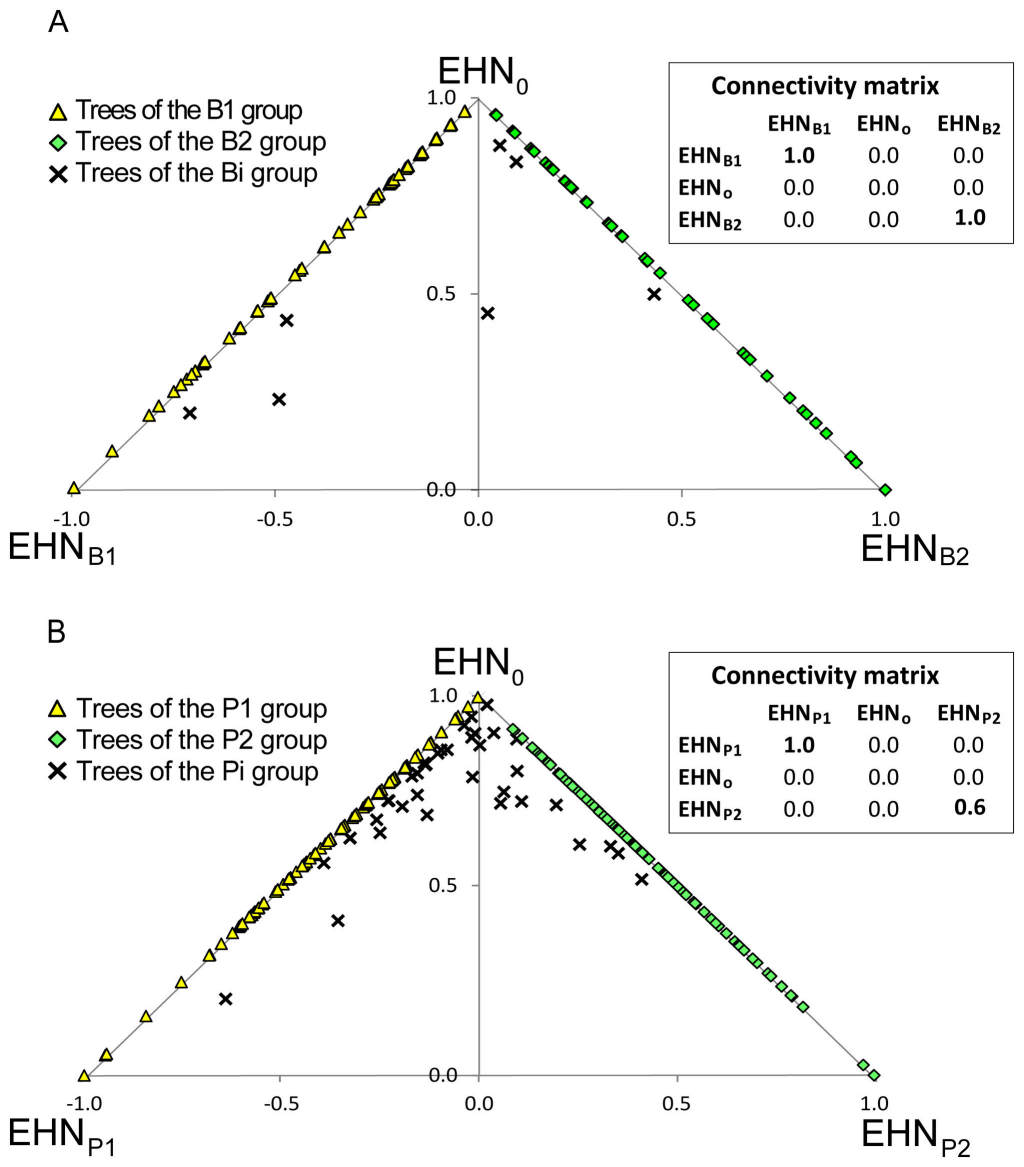
Triangular representation of the nodes of (A) the mating network and (B) the relatedness network, indicating the mixture of EHNs (i.e. Extremal Hypothetical Nodes) for each node according to C-SBM. In A, nodes that are on the edge between EHN_0_ and EHN_B1_ are classified in group B1 whilst nodes on the edge between EHN_0_ and EHN_B2_ are classified in group B2. Other individuals are classified as intermediates (group Bi). In B, nodes that are on the edge between EHN_0_ and EHN_P1_ are classified in group P1 whilst nodes on the edge between EHN_0_ and EHN_P2_ are classified in group P2. Other individuals are classified as intermediates (group Pi). Connectivity matrices for the EHNs are presented next to each triangular representation. Non-zero values are given in bold.

According to the AIC criterion, the best model for the mating network was the one with four EHNs, followed by the models with five and three EHNs (Figure S1 in File S1). We selected the model with three EHNs because the two other models highlighted the structure of the sampling design (Text S1 in File S1). According to the connectivity matrix for the EHNs (Figure 2A), EHN_0_ corresponds to a virtual node not connected to the whole network. This EHN, which is systematically present in the network models produced by C-SBM [24], makes it possible to take into account the variation in the number of links attached to the nodes of the real network. The two other EHNs, called EHN_B1_ and EHN_B2_, were strongly connected within themselves and were not connected to the other EHNs.

**Figure 3:**
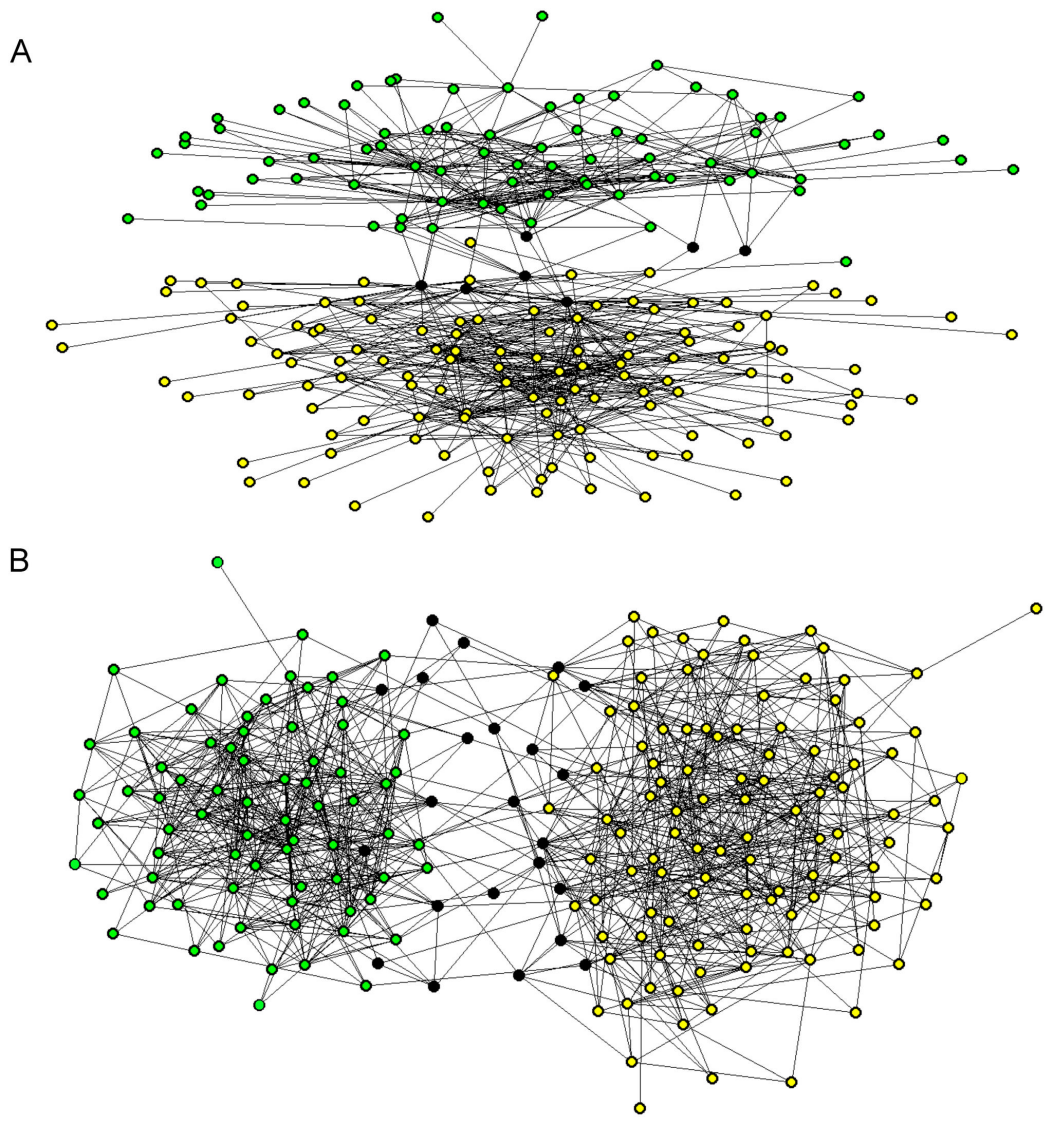
Graphical representation of (A) the mating network and (B) the relatedness network, using the software PAJECK with the following parameters: Draw/Layout/Energy/Kamada-Kawaï/Separate Components. Individuals classified into the B1 group (in A) or the P1 group (in B) are shown in green, individuals belonging to the B2 group (in A) or the P2 group (in B) are shown in yellow, and intermediate individuals are shown in black.

The nodes of the mating network (each corresponding to an individual) were then represented in a triangle, with one EHN at each point (Figure 2A). The higher the proportion of a given EHN in the mixture of a node, the closer the node was to this EHN in the triangle. According to the connectivity matrix for the EHNs (Figure 2A), the nodes that had a high proportion of EHN_0_ in their mixture were weakly connected to the mating network. The nodes that had a high proportion of EHN_B1_ in their mixture belonged to a group of nodes strongly connected to each other and weakly connected to nodes with a high proportion of EHN_B2_. Conversely, the nodes that had a high proportion of EHN_B2_ in their mixture belonged to a group of nodes strongly connected to each other and weakly connected to nodes with a high proportion of EHN_B1_. There were, therefore, two groups of adult trees in the mating network within which mating events were frequent and between which mating events were rare. The graphical representation of the network confirmed this result (Figure 3A). According to the relaxed interpretation of the biological species concept, these two groups of individuals should correspond to two biological species (Figure 1B).

In order to assign the individuals to the two species, we classified the nodes of the mating network according to their relative proportions of EHN_B1_ and EHN_B2_. We assumed that an individual belonged to species B1 if the corresponding node was a mixture of EHN_0_ and EHN_B1_ and only of these two nodes. Conversely, we assumed that an individual belonged to species B2 if the corresponding node was a mixture of EHN_0_ and EHN_B2_. Other individuals were classified as being reproductively intermediate (group Bi). In the triangular representation (Figure 2A), individuals assigned to species B1 were on the edge between EHN_0_ and EHN_B1_ (n=78 individuals) whilst individuals assigned to species B2 were on the edge between EHN_0_ and EHN_B2_ (n=121 individuals). Intermediate individuals were within the triangle (n=7 individuals). The three groups are shown in different colors in the network representation (Figure 3A).

### Species Delimitation based on Relatedness

According to the AIC criterion, the optimal number of EHNs in the relatedness network was six. Models with three, four, five and seven EHNs were also good models (Figure S2 in File S1). As we did not find any satisfactory way to identify the best model (Text S2 in File S1), we selected the model with three EHNs to facilitate a comparison between the relatedness network structure and the mating network structure. According to the connectivity matrix for the EHNs (Figure 2B), EHN_0_ corresponded to a virtual node not connected to the whole network. The two other EHNs, called EHN_P1_ and EHN_P2_, were strongly connected within themselves and were not connected to the other EHNs. Like the mating network, the individuals were, therefore, classified into three groups called P1, P2 and Pi. Group P1 (n=70 individuals located on the edge between EHN_0_ and EHN_P1_ in the triangular representation; Figure 2B) and group P2 (n=108 individuals located on the edge between EHN_0_ and EHN_P2_; Figure 2B) comprised individuals with high within-group and low between-group degrees of relatedness. The third group Pi (n=28 individuals located within the triangle; Figure 2B) included trees related to both P1 and P2 individuals and trees with few relatives. The three groups are shown in different colors in the network representation (Figure 3B).

### Species Delimitation based on Morphology and Multilocus Genotypes

The morphological similarity criterion has previously been used by Bacilieri *et al.* [26] to identify all trees from the study site. Based on their results, we assigned the individuals to two pure morphological groups (called M1 and M2 in this study and corresponding to *Q* *. robur* and *Q* *. petraea* , respectively) and to a morphologically intermediate class (called Mi). Guichoux *et al.* [23] used genotypic similarity as a criterion to assign the trees of the study site to species. Based on their results, we classified the adult trees in two purebred groups (hereafter called G1 and G2) and one genetically intermediate class (Gi).

### Congruence between the Four Methods of Species Delimitation

**Figure 4:**
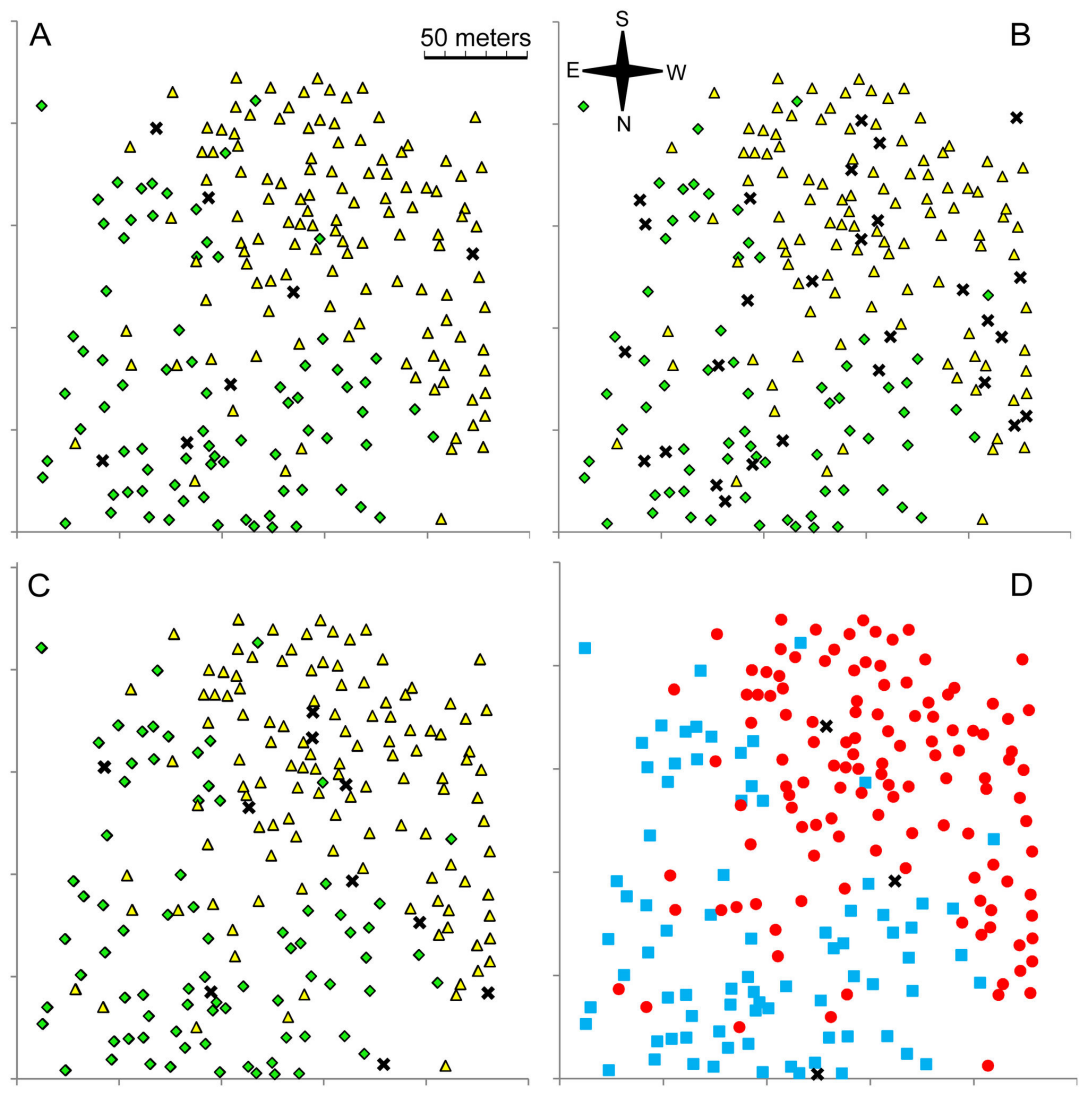
Species boundaries based on interfertility (**A**)**, relatedness **(**B**)**, genotypic similarity **(**C**) **and morphological similarity **(**D**) **criteria, represented on the map of the stand.** In A, B and C, individuals classified into the B1, P1 or G1 species, respectively, are represented by yellow triangles. Individuals classified into the B2, P2 or G2 species are represented by green diamonds. Intermediate individuals are represented by black crosses. In D, individuals classified into M1 are shown in red, individuals classified into M2 in blue and morphologically intermediate individuals are indicated by black crosses. Individuals of the M1 group are assigned to *Q. robur* and individuals of the M2 group to *Q. petraea* on the basis of current taxonomical practices.

In order to assess the congruence between the four methods of species delimitation, we compared the spatial distribution of the three groups of individuals identified with each method. The species boundaries are very similar (Figure 4). Among the 206 adult trees included in the mating network and in the relatedness network, there were 97 trees classified consistently in the B1, P1, G1 and M1 groups and 63 trees classified consistently in the B2, P2, G2 and M2 groups. We therefore re-named groups B1, P1, G1 and M1 *Q* *. robur* and groups B2, P2, G2 and M2 *Q* *. petraea* . Based on this classification, there were only four species inversions associated with the delimitation methods (Table S1 in File S1). Among the 206 adult trees, 42 were classified as intermediates according to at least one method. Surprisingly, no individual was classified as intermediate according to all four methods. Therefore, 91% of the discrepancies between the four methods were caused by assignments to the intermediate class (Figure S3 and Table S1 in File S1).

There were nine discrepancies between the individual assignments according to the genotypic and morphological similarity criteria on the one hand and the interfertility criterion on the other hand. We investigated whether the biotic environment of the individuals might account for these discrepancies. Our hypothesis is that the neighborhood of each tree influences its mating system and might thus influence its assignment to species based on the interfertility criterion, whereas it would hardly affect its assignment to species based on the genotypic and morphological criteria. We therefore examined the neighborhood of each tree for which the assignment to species based on genotypic and morphological similarity criteria were congruent (N= 192). For each tree, we calculated the proportion of allospecific neighbors within a radius of 69m (corresponding to the average distance of pollen dispersal within stand for *Q* *. petraea* , the species with the smallest dispersal ability [22]). We found, by performing a logistic regression, that the proportion of allospecific neighbors had a significant effect on the congruence between the individual assignments according to the genotypic and morphological similarity criteria on the one hand and the interfertility criterion on the other hand (χ^2^=6.5, df=1, p-value=0.01). The individuals with congruent assignments had fewer allospecific neighbors on average (29%, versus 51% for individuals with incongruent assignments). Hence, individual species assignments based on the interfertility criterion were environment-dependent.

## Discussion

To our knowledge, this is the first time that the interfertility criterion is used successfully to delimit species under natural conditions. The analysis of a network of mating events between pairs of adult trees, constructed on the basis of a powerful paternity analysis of a large number of seedlings produced under natural conditions, allowed us to identify two groups of interfertile individuals with only a few mating events between groups. The two groups that were delimited, corresponding to two species according to a relaxed interpretation of the biological species concept (Figure 1B), were closely congruent with those obtained previously using morphological and genotypic similarity as criteria for species delimitation [23,26]. Indeed, 88% of the individuals were classified consistently according to the interfertility, morphological similarity and genotypic similarity criteria. Our results do not support earlier claims that the interfertility criterion cannot be applied in the field (e.g. [14,15]), particularly in the genus *Quercus* [27]. They show instead that the analysis of mating networks can be used for delimiting species according to the biological species concept, as first suggested by Sokal and Crovello [14].

However this method of species delimitation has two main drawbacks. First, adequate network data are difficult to assemble. In our study we performed a paternity analysis on as many as 3046 offspring produced by 51 mothers in order to construct the mating network for adult trees. Despite the very large number of offspring, our network data did not allow us to assign all the individuals in the forest stand to species. Not all individuals sired offspring and some sired too few offspring to be reliably connected to the network. For example, three of the individuals whose assignment based on the interfertility criterion differed from that based on the three other criteria were represented by a single offspring in the progeny test. They were thus connected to the mating network through just a single link. Second, the sampling design may generate some heterogeneity in the network structure that blurs the biological heterogeneity caused by the existence of different species. This happened in our network data because we harvested the offspring of only 20% of the trees in the stand. The harvested trees (i.e. mother-trees), therefore, had more links in the mating network than the other trees. To solve both problems, one would have to harvest seeds from all the individuals in the stand, assuming that all of them produced seeds. In principle, this goal could be achieved with our biological system by extending sampling over multiple years, because oak species are perennial and monoecious. However this would be impossible for annual or dioecious species. Another possibility to reduce the noise caused by sampling would be to introduce the sampling structure as a covariate in the statistical model (e.g. [28]). Unfortunately, the Continuous Stochastic Block Model [24], which was selected for this study because it allows modeling continuous variations in the connectivity properties of the nodes, does not currently allow the incorporation of covariates.

Our results further show that the analysis of the network of contemporary relatedness relationships is a relevant method for delimiting species. The two groups found in our study might be interpreted as corresponding to two different ‘phylogenetic species’ [29], if phylogenetic relationships are considered in a broad sense so as to include contemporary pedigree relationships. Methods of species delimitation derived from the phylogenetic species concept have almost exclusively focused on deep ancestry using tree-based phylogenetic methods (reviewed in 30, but see 31). These methods are not well-suited for delimiting hybridizing species because horizontal gene transfers between species, caused by hybridization and subsequent backcrossing events, produce conflicts between gene trees and species trees [32,33]. Compared to data on mating events, data on relatedness were easier to acquire and there was no sampling issue. The analysis of the relatedness network revealed two groups of individuals with high within-group and low between-group degrees of relatedness. These two groups were highly congruent with those obtained using interfertility, morphological similarity and genotypic similarity as criteria, indicating that the analysis of relatedness networks may have potential for species delimitation. However, this method also has some drawbacks: the best model had five groups of related individuals and we did not find any hypothesis accounting for their origin; the number of species should thus be known in advance in order to apply this method.

By comparing the results obtained with the four criteria used for species delimitation (i.e. interfertility, relatedness relationships, morphological or genotypic similarities), we showed that the species boundaries were largely congruent across methods of species delimitation. Our analyses confirmed the existence of two groups of individuals that were both morphologically and genetically differentiated. We also showed that the individuals of each group preferentially mated and were more related with each other than with individuals from the other group. Therefore, there were two ‘evolutionary lineages’ in the studied stand. The Lineage Species Concept introduced by Simpson [34,35], then taken up by Wiley [36] and de Queiroz [16,37,38], focuses on the question of congruence among methods of species delimitation. For these authors, modern species concepts (e.g. morphological, phylogenetic, genotypic and biological) assimilate, explicitly or implicitly, species ‘to separately evolving (segments of) metapopulation lineages’ and are thus all by-products of the lineage species concept [16,17]. This should account for the high degree of congruence among species delimitation methods.

Another important result of this comparison is that, irrespective of the criterion used for delimiting species, we found intermediate individuals that had features of both species. Interestingly, the individuals classified as intermediates often differed across methods. In particular, no individual was consistently classified as intermediate according to all four methods. These discrepancies might be explained by the thresholds that were chosen empirically to delimit purebred species and by data quality problems. As mentioned above, examining more offspring per parent tree may improve species delimitation based on the interfertility criterion. Similarly, a greater number of molecular markers [39] may improve methods of species delimitation based on the genotypic and relatedness criteria. Likewise, a larger number of morphological markers [26] may improve morphological species delimitation. However, we believe that these discrepancies may also reflect a biological reality. Indeed, as shown in other studies [40–42], including in oaks [22,43], species relative abundance affects hybridization rate. An individual tends to reproduce with its neighbours. If it is surrounded by numerous allospecifics and few conspecifics (e.g. [22,42]), this can result in much hybridization. Such an individual will tend to be assigned to another species or to a reproductively intermediate class, according to methods of delimitation based on interfertility. Therefore, we expect some discrepancies in species assignments between methods based on environment-dependent criteria (such as that based on the interfertility criterion) and methods based on environment-independent criteria (such as that based on the genotypic similarity criterion). Because of these fundamental differences among methods, it is impossible to compute a reference dataset that would give the correct assignment of each individual. Our results thus cannot be used to identify one method of species delimitation that would produce more reliable assignments than the others. Instead, our results show that different methods of species delimitation produce slightly different results when applied to real biological data.

## Conclusion

Our results confirmed the existence of two differentiated groups of individuals at the study site, corresponding to two species: *Quercus* *robur* and *Q* *. petraea* . However, depending on the criterion used for assigning individuals to species (i.e. interfertility, relatedness, morphological or genotypic similarities), the boundary between species was not exactly the same. Most of the differences stem from assignment of individuals to an intermediate category. This finding illustrates the continuous nature of variation between species. The model we used, which belongs to a category called ‘grade of membership models’ (reviewed in 10) is appropriate for synthesizing continuous (but not uniform) variations in biological diversity. However, to get closer to the species concepts, which generally define species as groups of individuals, we finally classified the individuals into non-overlapping groups. Our approach, therefore, illustrates the influence of concepts on our (mis)representation of species and on our understanding of biological diversity. Frost and Hillis [44], as well as Mayr [45], proposed defining species as ‘a whole’ instead of as a group of individuals. According to our study, species could also be defined as an ‘extreme point’ to which individuals are more or less close, thus allowing the possibility of an individual being a mixture of two different species.

## Materials and Methods

### Species Delimitation based on Interfertillity

To construct the mating network for the adult trees, we made use of a progeny test involving 3046 offspring resulting from open pollination, harvested from 51 mother-trees distributed across the entire stand (Figure S4 in File S1). A paternity analysis was conducted [22] by genotyping all the offspring from the test and all the adults trees for which DNA was available, using 12 multiplexed microsatellite (SSR) markers developed by Guichoux *et al.* [46]. According to the paternity analysis, 1575 offspring had only one possible father in the stand, 54 offspring had several potential fathers in the stand and 1417 offspring had no father in the stand [22]. Based on the offspring for which only a single father was found, we identified 198 father-trees in the stand. These trees included 43 trees that were also mothers, because oak trees are monoecious. Based on these results, we reconstructed 1629 mating events between 206 adult trees within the stand. These mating events allowed us to identify 751 couples of trees that mated at least once, indicating that they were interfertile under natural conditions. These data were represented by an undirected and unweighted network in which each of the 206 nodes corresponded to an adult tree and each of the 751 links corresponded to at least one mating event between two trees.

Then, the network was modeled with C-SBM [24]. The parameters of the model are the connectivity coefficients between the EHNs and the coefficients of the mixture of EHNs for each node of the real network. For each possible number of EHNs, the parameters of the model were inferred by the maximum likelihood method, derived using the MATLAB program C-Mixnet (available at http://www.agroparistech.fr/mia/doku.php?id=productions:logiciels/). Then, the optimal number of EHNs in the network was determined by using the AIC criterion [24]. The results were visualized with the software pajek [47].

### Species Delimitation based on Relatedness

In order to build the relatedness network, we estimated the relatedness of the 206 adult trees included in the mating network. The estimation was performed with the software COANCESTRY [48], which offers seven different estimators of relatedness. As recommended by Wang [48], we used the 1629 offspring for which both parents were known to determine the most suitable estimator. The triadic likelihood estimator (denoted TrioML in COANCESTRY [49]) was selected because it produced relatedness values closest to zero for unrelated offspring, closest to 0.25 for half-sibs and closest to 0.5 for full-sibs. With this estimator, the highest relatedness value between two unrelated offspring was 0.22. We therefore treated 0.22 as a threshold: trees whose relatedness value was higher than this were considered to be related individuals and the other trees were considered to be unrelated. The relatedness relationships were then represented by an unweighted and undirected network with 206 nodes, each corresponding to an adult tree, and 1078 links connecting the individuals considered to be related. As in the case of the mating network, we modeled the network structure using C-SBM [24] and we visualized the results with the software pajek [47].

### Species Delimitation based on Morphology

The morphological similarity criterion has previously been used by Bacilieri *et al.* [26] to identify all trees from the study site. These authors performed a factorial discriminant analysis (FDA) based on 31 leaf morphological traits to delimit the species. Their study revealed the presence of two groups of individuals differing in their morphology. The first axis of the FDA accounted for 33% of the total variance and was highly correlated to the morphological markers traditionally used by taxonomists to distinguish *Q* *. robur* from *Q* *. petraea* . The distribution of the individuals along this axis was used to assign, graphically, the individuals to two pure morphological groups (called M1 and M2 in this study and corresponding to *Q* *. robur* and *Q* *. petraea* respectively) and to a morphologically intermediate class (called Mi). Among the 206 adult trees included in the mating and relatedness networks, 123 trees were assigned to M1, 80 to M2 and 3 to Mi (Figure S5 in File S1).

### Species Delimitation based on Multilocus Genotypes

Guichoux *et al.* [23] used genotypic similarity as a criterion to assign the trees of the study site to species. These authors genotyped the adult trees with the multiplex of 12 SSRs developed by Guichoux *et al.* [46] and with a chip of 262 single-nucleotide polymorphisms (SNP) enriched with markers highly differentiated between species [23]. They used the software structure [50] to group the individuals into genotypic clusters but did not formally determine the optimal number of genotypic clusters in the stand before performing the clustering. Here we used the ΔK statistic [51] to identify the number of genetically different groups. The optimal number of clusters was two (Figure S6 in File S1), as previously assumed by Guichoux *et al.* [23]. The adult trees were therefore classified in two purebred groups and one genetically intermediate class. Among the 206 adult trees included in the mating and relatedness networks, 78 trees were assigned to the first purebred group (hereafter called G1), 118 to the second purebred group (G2) and 10 to the genetically intermediate class (Gi) (Figure S7 in File S1).

## Supporting Information

File S1 (PDF)
