# Supplementary material for: Putting the Biological Species Concept to the Test: Using Mating Networks to Delimit Species
Source: PLoS One. 2013 Jun 20;8(6):e68267. doi: 10.1371/journal.pone.0068267 (PMC3688613; doi:10.1371/journal.pone.0068267)
Supplement: File S1 — (PDF) [file pone.0068267.s001.pdf]

SUPPLEMENTARY INFORMATION

- **Figure S1:** Optimal number of EHNs in the mating network according to the AIC criterion
- **Text S1:** Effect of sampling design on the heterogeneity of the mating network
- **Figure S2:** Optimal number of EHNs in the relatedness network according to the AIC criterion
- **Text S2:** Effect of spatial structure of the trees on the heterogeneity of the relatedness network
- **Table S1:** Comparison of individual assignments to species based on interfertility, relatedness, morphological and genotypic similarities criteria.
- **Figure S3:** Percentage of individuals assigned to *Q. robur*, *Q. petraea* and the intermediate class according to one criterion only, or two, three and four criteria consistently.
- **Figure S4:** Map of the oak stand
- **Figure S5:** Morphological species delimitation
- **Figure S6**: Optimal number of genotypic clusters according to Evanno *et al.* criterion
- **Figure S7:** Genotypic species delimitation
- **References**

<u>Figure S1:</u> Optimal number of EHNs in the mating network according to the AIC criterion. The highest AIC values, circled with a red dotted line, correspond to the best models. The optimal number of EHNs is 4 (AIC=-8345.5), followed by 5 (AIC=-8443) and then 3 (AIC=-8702.3).

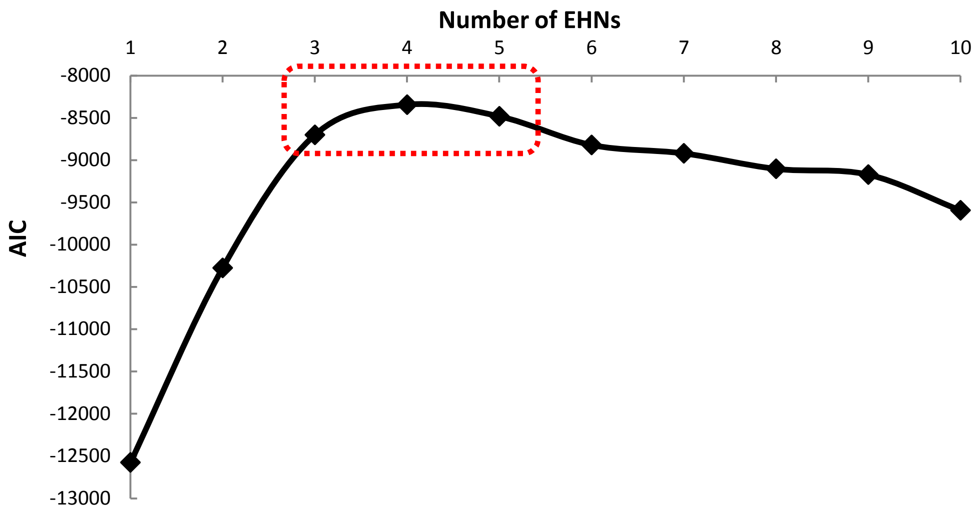

Text S1: Effect of the sampling design on the heterogeneity of the mating network

There are two key elements for interpreting the heterogeneity of a real network modeled with C-SBM:

- The connectivity matrix between the EHNs
- The mixture of EHNs for each node of the network

Each element a_{ql} of the connectivity matrix A between the EHNs corresponds to the probability that there is a link between EHN_q and EHN_l. There is always one EHN, called EHN_0, which is not connected to itself and not connected to the other EHNs. In the mating network, the other EHNs, called $EHN_{Bj(1 \leq j \leq k-1)}$, are strongly connected with themselves and not connected with the other EHNs.

k=3

	EHN_{B1}	EHN_0	EHN_{B2}
EHN_{B1}	**1.0**	0.0	0.0
EHN_0	0.0	0.0	0.0
EHN_{B2}	0.0	0.0	**1.0**

k=4

	EHN_{B1}	EHN_0	EHN_{B2}	EHN_{B3}
EHN_{B1}	**1.0**	0.0	0.0	0.0
EHN_0	0.0	0.0	0.0	0.0
EHN_{B2}	0.0	0.0	**1.0**	0.0
EHN_{B3}	0.0	0.0	0.0	**1.0**

k =5

	EHN_{B1}	EHN_0	EHN_{B2}	EHN_{B3}	EHN_{B4}
EHN_{B1}	**1.0**	0.0	0.0	0.0	0.0
EHN_0	0.0	0.0	0.0	0.0	0.0
EHN_{B2}	0.0	0.0	**1.0**	0.0	0.0
EHN_{B3}	0.0	0.0	0.0	**1.0**	0.0
EHN_{B4}	0.0	0.0	0.0	0.0	**1.0**

Connectivity matrices between the EHNs in the mating network, as a function of the number k of EHNs. The connectivity properties of EHN_0 are highlighted in grey. Non-zero values are in bold.

The more a node has a high proportion of a given EHN in the mixture, the more its connectivity properties resemble to those of this EHN. Therefore, the nodes with a high proportion of EHN_0 in the mixture are lowly connected to the network (i.e. they have a low degree). The nodes with a high proportion of one of the $EHN_{Bj(1 \leq j \leq k-1)}$ in the mixture belong to a group of nodes strongly connected between them, and lowly connected with nodes of other groups (i.e. groups of interbreeding individuals, reproductively isolated from other groups). In order to delimit species based on the

interfertility criterion, we therefore grouped together the nodes according to the proportion of each $EHN_{Bj(1≤j≤k-1)}$ in the mixture. We assumed that a node belongs to group B_j if it is a mixture between EHN_0 and EHN_{Bj}, and only between these two EHNs. Other individuals were classified as intermediate. We then analyzed the composition of the groups as a function of the sampling design.

k=3

	Unsampled	Sampled
**B_1**	56	22
**B_2**	96	25

k=4

	Unsampled	Sampled
**B_1**	97	**0**
**B_2**	**0**	8
**B_3**	54	22

k=5

	Unsampled	Sampled
**B_1**	18	4
**B_2**	**0**	3
**B_3**	52	**0**
**B_4**	48	21

Number of sampled and unsampled trees in each group as a function of k. The groups having only one type of tree are highlighted in grey.

In the models with 4 and 5 EHNs, one group was composed of samples trees only and another group was composed of unsampled trees only, indicating that the heterogeneous structure of the mating network is partly accounted for by the sampling design. Describing the heterogeneity due to sampling was not the purpose of our study so we selected the model with 3 EHNs, in which groups were independent from the sampling design.

Figure S2: Optimal number of EHNs in the relatedness network according to the AIC criterion. The highest AIC values, circled with a red dotted line, correspond to the best models. The optimal number of EHNs is six (AIC = -20759), then seven (AIC = -20975), four (AIC = -21006), five (AIC = -21138) and finally three (AIC = -21210).

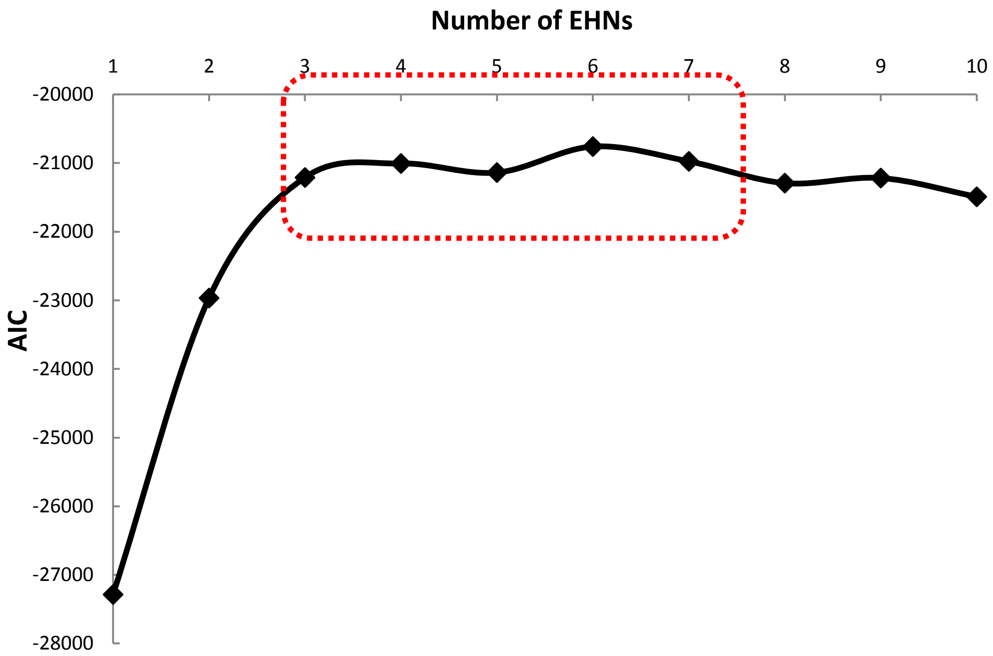

<u>Text S2</u>: Effect of the spatial structure of the trees on the heterogeneity of the relatness network

According to the AIC criterion, the best model had six EHNs (Figure S6). According to the connectivity matrix between the EHNs, five of these EHNs (called EHN_{Pj} with $1 \leq j \leq 5$) were highly connected with themselves and not connected with the other EHNs.

	**k=6**					
	**EHN_0**	**EHN_{p1}**	**EHN_{p2}**	**EHN_{p3}**	**EHN_{p4}**	**EHN_{p5}**
**EHN_0**	0.0	0.0	0.0	0.0	0.0	0.0
**EHN_{p1}**	0.0	0.8	0.0	0.0	0.0	0.0
**EHN_{p2}**	0.0	0.0	1.0	0.0	0.0	0.0
**EHN_{p3}**	0.0	0.0	0.0	1.0	0.0	0.0
**EHN_{p4}**	0.0	0.0	0.0	0.0	1.0	0.0
**EHN_{p5}**	0.0	0.0	0.0	0.0	0.0	1.0

<u>Connectivity matrix between the EHNs in the relatedness network, for k=6</u>

Following the same method than for the mating network (Appendix S1), we thus classified the individuals into five groups of related individuals (called P1 to P5) and one group of intermediate individuals (called Pi). The limited dispersal of pollen grains and seeds in the studied oak species [1,2,3] might have generated a spatial structure in the relatedness relationships, with local subgroups of individuals strongly related to each other. Therefore, we investigated whether the five groups of related individuals corresponded to geographical groups. However, this was not the case (see below).

Map of the oak stand with individual assignments to groups based on the best model for the relatedness network

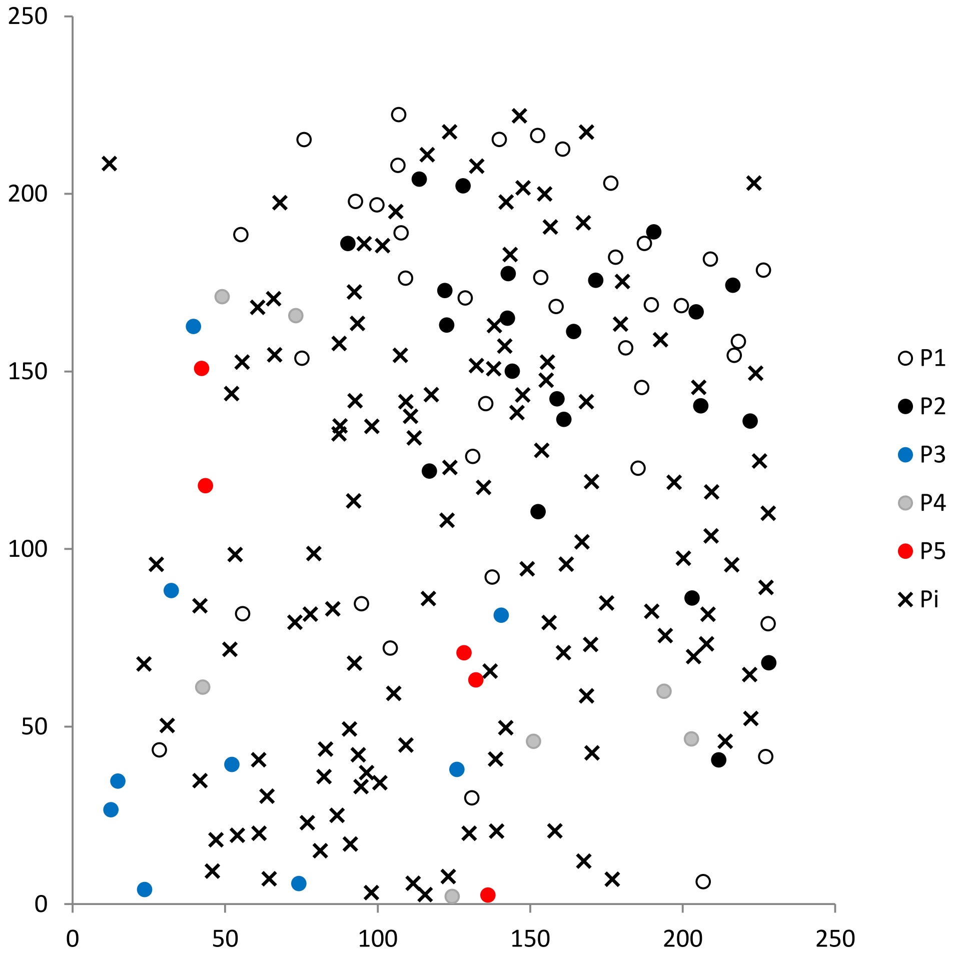

Table S1: Comparison of the individual assignments to species based on interfertility, relatedness, morphological and genotypic similarities criteria

Assignment based on the four criteria (i.e. interfertility, relatedness, genotypic and morphological similarities)	Number of individuals
**The four criteria are in agreement**	**160**
Qr Qr Qr Qr	97
Qp Qp Qp Qp	63
I I I I	0
**Three criteria are in agreement**	**42**
Qr Qr Qr I	26
Qp Qp Qp I	11
Qp Qp Qp Qr	2
Qr Qr Qr Qp	2
I I I Qr	1
**Two criteria are in agreement**	**4**
Qp Qp I I	2
Qr Qr I I	2

Qr: *Quercus robur*, Qp: *Quercus petraea*, I: intermediate.

<u>Figure S3:</u> The percentage of individuals assigned to *Q. petraea* (Qp), the intermediate class (I), and *Q. robur* (Qr) according to one criterion only (blue), or two (green), three (white) and four criteria (orange) consistently, out of all individuals assigned to this category by at least one criterion. The total number of individuals assigned to each category by at least one criterion is indicated above the bars.

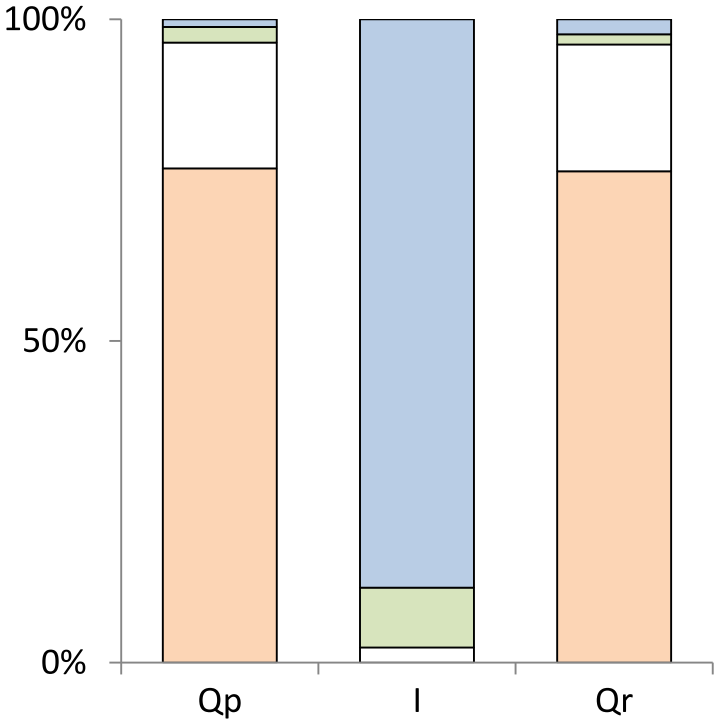

<u>Figure S4:</u> Map of the oak stand. The stand was composed of 298 adult trees, among which 206 were genotyped and assigned to species by Guichoux *et al.* [4]. Trees assigned to the *Quercus robur* species by Guichoux *et al.* [4] are represented by grey diamonds and trees that were assigned to the *Q. petraea* species are represented by black squares. Trees considered as hybrid trees by Guichoux *et al.* [4] are represented by white triangles. Trees on which acorns were sampled to set up the progeny test are circled.

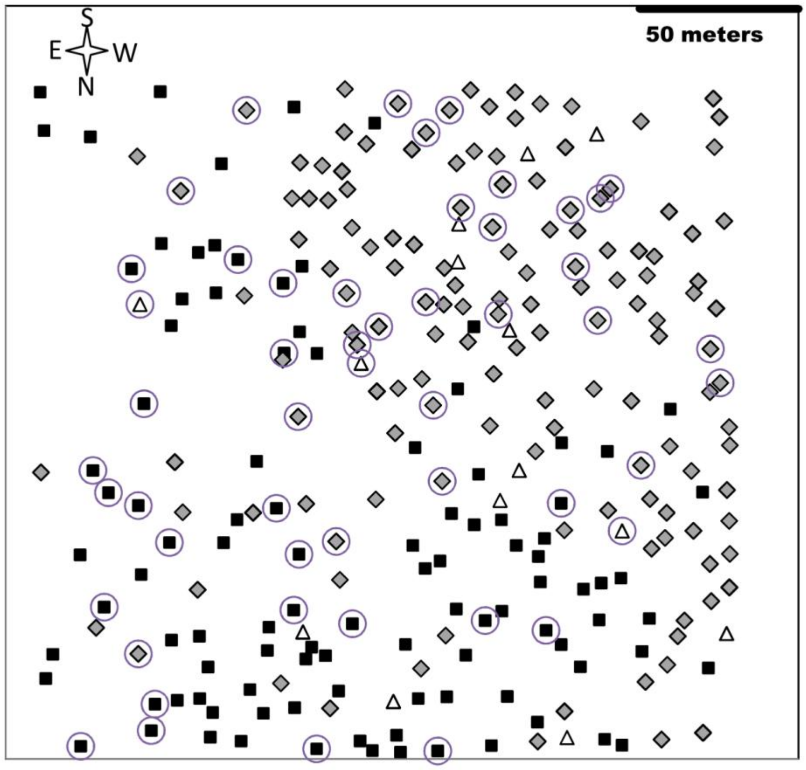

<u>Figure S5:</u> Morphological Species delimitation. The 206 oak trees were ordered on the x-axis as a function of their value on the first axis of the Factorial Discriminant Analysis (FDA) performed by Bacilieri *et al.* [5], which was based on 31 morphological traits of leaves. Individuals were then graphically classified into two groups (M1 and M2) and an intermediate class. Individuals assigned to M1 are represented in red, those assigned to M2 are represented in blue, and trees with an intermediate morphology are represented in black. Leaf morphology reveals that individuals in M1 group are *Q. robur* and in M2 group are *Q. petraea* individuals. Morphological data can be found at http://bioinfo.orleans.inra.fr/TreePop/tmp/export_20121002141319506ada5f6da21.txt.

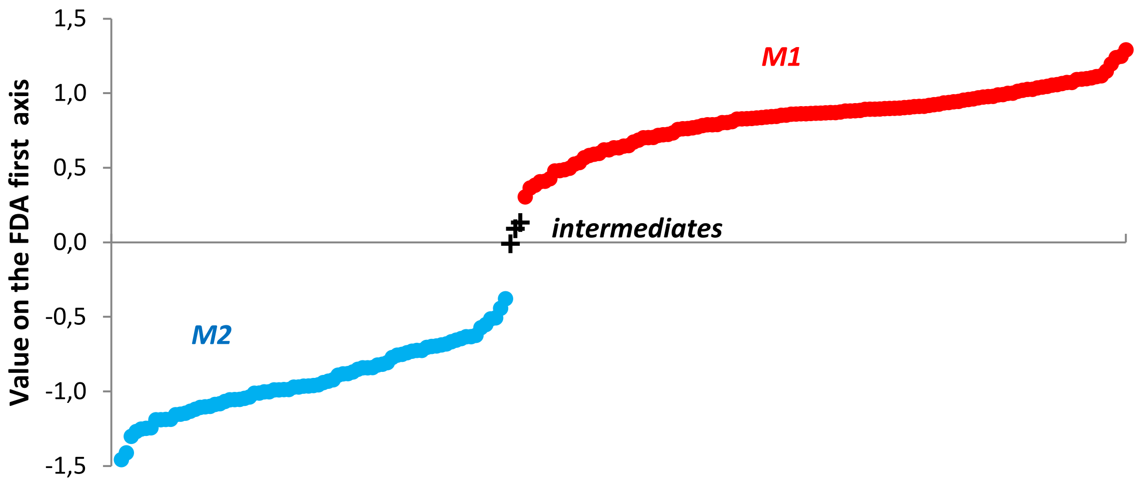

Figure S6: **Optimal number of genotypic clusters according to the Evanno's criterion [6],** calculated by running STRUCTURE with the following parameters: 50000 burning, 50000 Markov chain with admixture, number of genotypic clusters (k) varying from 1 to 6 with five repetitions for each k values. The optimal number of clusters, indicated in red, is given by the highest Δk value. Microsatellite data can be found in the Dryad data repository at http://datadryad.org, doi:10.5061/dryad.n50b4.

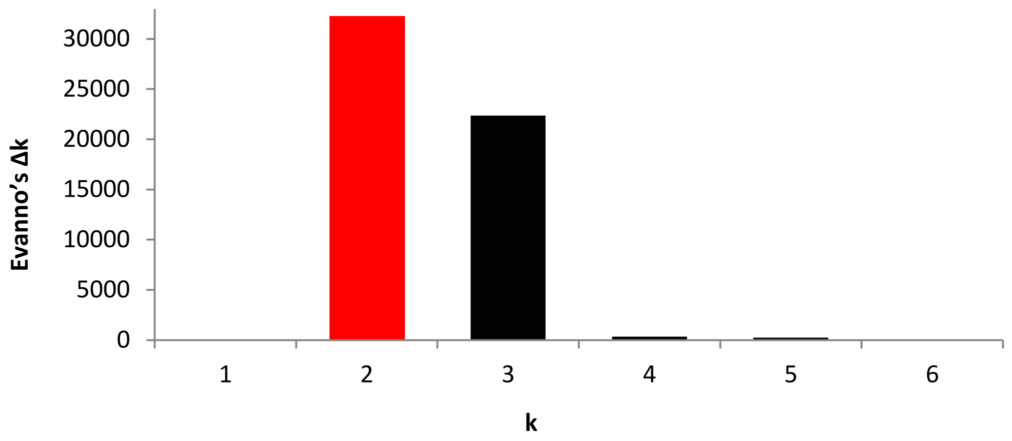

<u>Figure S7:</u> Genotypic species delimitation. The 206 individuals were ordered on the x-axis as a function of the admixture degree to the G1 group, obtained by Guichoux *et al.* [4]. Individuals were classified into two groups and an intermediate class, by using the same thresholds than in Guichoux *et al.* [4]. The lower and the higher thresholds represented by dotted grey lines, equal respectively 0.125 and 0.875. Individuals assigned to the G1 group are symbolized by white triangles, those classified into the G2 group by black diamonds and intermediate individuals (Gi group) are represented by black crosses. These three groups were respectively called *Q. petraea*, *Q. robur* and hybrids by Guichoux *et al.* [4]. In the present study we preferred naming them G1, G2 and Gi, in order to differentiate them from the groups obtained through morphological data analysis.

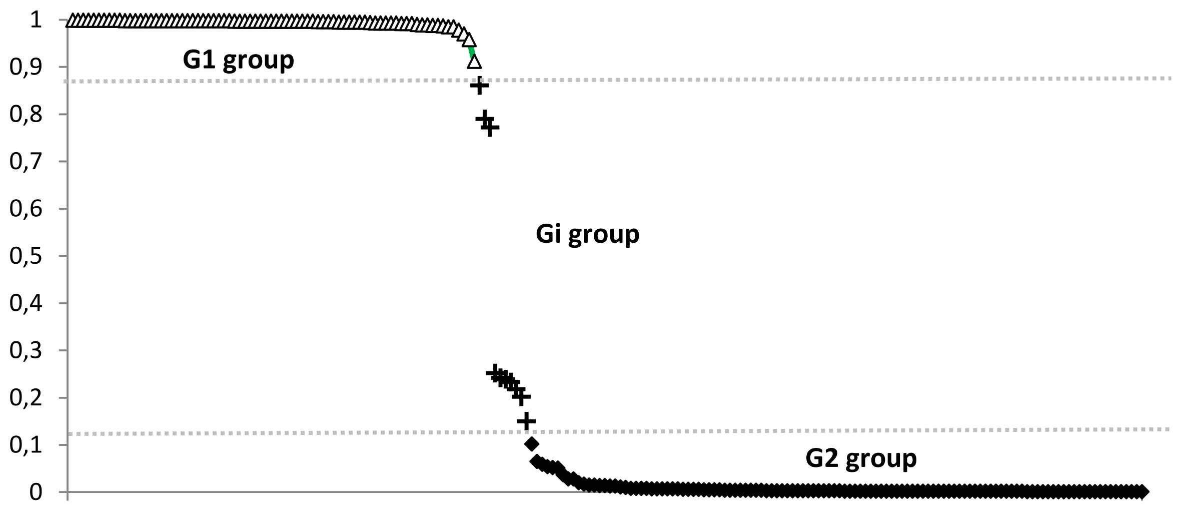

REFERENCES:

1. Lagache L, Klein EK, Guichoux E, Petit RJ (2012) Fine-scale environmental control of hybridization in oaks. Molecular Ecology: *in press*.
2. Jensen J, Larsen A, Nielsen LR, Cottrell J (2009) Hybridization between *Quercus robur* and *Q. petraea* in a mixed oak stand in Denmark. *Annals of Forest Science* 66.
3. Pons J, Pausas J (2007) Acorn dispersal estimated by radio-tracking. Oecologia 153: 903-911.
4. Guichoux E, Garnier-Géré P, Lagache L, Lang T, Bourry C, et al. (2012) Outlier loci highlight the direction of introgression in oaks. Molecular Ecology: *in press (MEC-12-0795.R0791)*.
5. Bacilieri R, Ducousso A, Kremer A (1996) Comparison of morphological characters and molecular markers for the analysis of hybridization in sessile and pedunculate oak. *Annales des Sciences Forestières* 53: 79-91.
6. Evanno G, Regnaut S, Goudet J (2005) Detecting the number of clusters of individuals using the software structure: a simulation study. Molecular Ecology 14: 2611-2620.
